# The Dichotomy of Tumor Exosomes (TEX) in Cancer Immunity: Is It All in the ConTEXt?

**DOI:** 10.3390/vaccines3041019

**Published:** 2015-12-17

**Authors:** Katherine E. Kunigelis, Michael W. Graner

**Affiliations:** Department of Neurosurgery, Anschutz Medical Campus, University of Colorado Denver, Aurora, CO 80045, USA; E-Mail: katherine.kunigelis@ucdenver.edu

**Keywords:** exosomes/microvesicles/extracellular vesicles, tumor-derived exosomes (TEX), dendritic cell-derived exosomes (DEX), cancer vaccine, immune suppression, antigen presenting cells, T cells, B cells, natural killer cells

## Abstract

Exosomes are virus-sized nanoparticles (30–130 nm) formed intracellularly as intravesicular bodies/intralumenal vesicles within maturing endosomes (“multivesicular bodies”, MVBs). If MVBs fuse with the cell’s plasma membrane, the interior vesicles may be released extracellularly, and are termed “exosomes”. The protein cargo of exosomes consists of cytosolic, membrane, and extracellular proteins, along with membrane-derived lipids, and an extraordinary variety of nucleic acids. As such, exosomes reflect the status and identity of the parent cell, and are considered as tiny cellular surrogates. Because of this closely entwined relationship between exosome content and the source/status of the parental cell, conceivably exosomes could be used as vaccines against various pathologies, as they contain antigens associated with a given disease, e.g., cancer. Tumor-derived exosomes (TEX) have been shown to be potent anticancer vaccines in animal models, driving antigen-specific T and B cell responses, but much recent literature concerning TEX strongly places the vesicles as powerfully immunosuppressive. This dichotomy suggests that the context in which the immune system encounters TEX is critical in determining immune stimulation *versus* immunosuppression. Here, we review literature on both sides of this immune coin, and suggest that it may be time to revisit the concept of TEX as anticancer vaccines in clinical settings.

## 1. Introduction

Extracellular vesicles such as exosomes and microvesicles are now reasonably considered to be particularly important players in both the normal and pathological functions of cells, organs, and organisms [1]. Their roles in many biologic processes [2] are increasingly recognized, if not understood, including in immune function/dysfunction in relation to cancer [3,4,5]. Here, we will review some of the immune properties of exosomes/microvesicles in the context of cancer biology, where the vesicles may play dual roles in terms of immune stimulators (potentially having vaccine characteristics) and immune suppressors. We will argue that this dichotomy is likely due to the context in which the immune system sees the vesicles, and those circumstances may be deciding factors in the direction taken by the immune response.

### Concerning Nomenclature

The terminology of secreted or extracellularly released nanovesicles is diverse, confusing, and perhaps, at times, whimsical. The literature on the topic yields dozens of different names for what are (now, tentatively) collectively called extracellular vesicles (EVs) [6]. Generally, EVs from typical eukaryotic cells are regarded as belonging to one of three categories [2]: “apoptotic bodies/blebs” coming from vacuolization or membranous vesiculation of cells dying from apoptotic processes (diameters ~1 μm to 5 μm); “microvesicles”, “microparticles” or “ectosomes” (and other terms) where vesicles are directly released or “shed” from the plasma membrane (diameters ~130 nm to 1 μm); and “exosomes”, referring specifically to the release of endosomally derived intralumenal vesicles of multivesicular bodies, as explained below (diameters ~30 to 130 nm) [7]. From an experimental viewpoint, different separation techniques tend to produce somewhat distinct vesicle types, but there is undoubtedly overlap, for instance at the high-end size of the “exosomes” and the low-end size of the “microvesicles”, in terms of protein markers and activities [8,9]. However, most of the literature we will review here regards vesicles with sizes and characteristics consistent with what are generally termed “exosomes”, and we will employ that term throughout the rest of this article.

## 2. Exosomes

**Figure 1:**
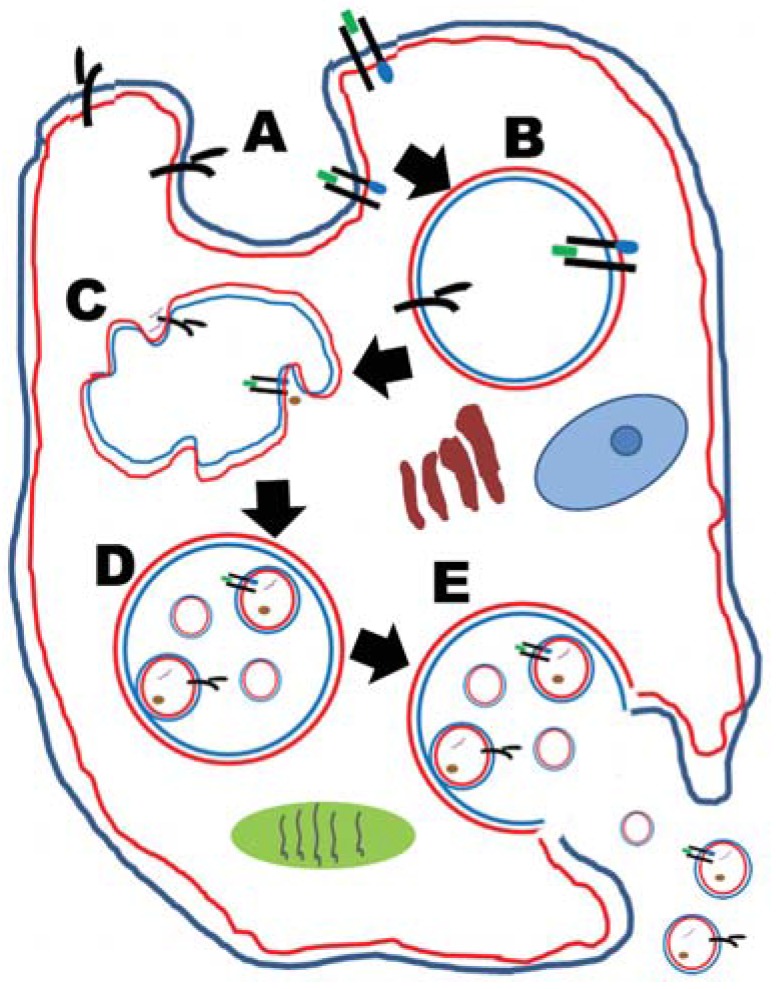
Exosome formation in a generic cell (nothing is drawn to scale). Exosomes are virus-sized (30–130 nm diameter) vesicles formed within the endosomal system that are released extracellularly. (**A**) shows an endocytic event (which could be clathrin- or caveolin-mediated, or independent; or phagocytic, or (macro) pinocytic). An early endosome is formed in (**B**); note the orientation of the membranes (blue or red outlines) and the nature of the former surface proteins (previously with extracellular domains that are now intralumenal within the endosome). In (**C**), membrane invaginations occur (largely through the efforts of the unshown ESCRT machinery) which push the transmembrane proteins into the endosomal lumen, and sequester cytosolic molecules (proteins: brown dots; nucleic acids: purple squiggle lines) inside intralumenal vesicles, as seen in (**D**). (**D**) represents a “multivesicular body” (MVB), a late endosomal intracellular vesicle with smaller vesicles within it. In (**E**), the MVB fuses with the plasma membrane to release the intralumenal vesicles outside the cell; these are now termed “exosomes”. Note that the membrane/membrane protein topography (as well as the cytosolic components) now recapitulates the original exterior and interior compartmentalization.

As stated, exosomes are nanoparticles (30–130 nm) formed by inward budding of endosomal membranes to form multivesicular bodies (MVBs). MVBs canonically converge to lysosomal degradation; a multitude of intracellular and extracellular conditions may instead drive MVBs to fuse with the cell plasma membrane to release their intralumenal vesicles as exosomes [10]. This is illustrated in Figure 1. One of their main functions is in intercellular communication, and as such, their contents represent their native environment, including proteins, lipids, and nucleic acids. They stand as surrogates of the status and identity of their cells of origin [11]. For this reason, exosomes procured from bodily fluids are proposed to be potentially extraordinary biomarkers of disease [12,13], and could be the chief diagnostic components of “liquid biopsy” [14,15,16].

Exosomes are secreted or released by essentially all cell types, certainly most immune and non-immune cells including macrophage, dendritic cells (DCs), B cells, T cells such as CD8+ cytotoxic T-lymphocytes (CTLs) and CD4+ T helper (T_H_) cells, platelets, mastocytes, fibroblasts, epithelial cells, as well as tumor cells. Exosomes participate in important biological functions, acting as a mode of communication between cells that does not require cell-cell contact, and can thus occur over significant distances [17]. This intercellular communication can be conferred by mediators that are expressed or displayed on the exosome surfaces, thus mimicking cell-cell interaction to some extent. Exosomes produced by both immune and non-immune cells have the ability to regulate immune responses [18], thus demonstrating an impact of exosomes in webs of intercellular, and inter-system, communications [19,20].

### 2.1. Tumor-Derived Exosomes (TEX)

Although the best-studied microvesicles in the cancer immunotherapy field are (arguably) exosomes, tumor-derived exosomes (TEX) are still associated with seemingly conflicting roles of immune stimulation as well as suppression of both tumor-specific and nonspecific immune responses [21,22]. As the induction of an active immune response to control or eliminate tumors remains an unfulfilled challenge, TEX have been proposed for a role in cancer immunotherapy through harnessing a source of cancer cell antigens for antitumor immune responses along with innate immune stimulation [23,24]. However, hope that the resulting TEX antigen-based vaccines are promising immunotherapeutic tools has been dampened by evidence showing that TEX interfere with immune response induction [5,25,26]. Here, we review both the immune stimulatory and suppressive effects reported for TEX as well as current directions for applied uses in cancer immunotherapy. However, some additional background on cancer vaccines and the roles exosomes have played (and continue to play) there may be insightful.

### 2.2. Dendritic Cell-Based Cancer Vaccines

Dendritic cells (DCs) are professional antigen-presenting cells (APCs) [27] that likely have functions and roles beyond their initial identity [28]. DCs are capable of uptake and digestion of potentially antigenic materials, along with the display of peptide antigens via MHC-I and MHC-II routes (to CD8+ and CD4+ T cells, respectively), and contributing appropriate co-stimulatory molecules to activate antigen-specific T cells via the “immunological synapse” [29]. Conceptually, one could generate and culture *ex vivo* DCs from a patient (or mouse), apply (“pulse”) sources of antigens to the DCs in culture, induce or allow the processing and presentation of antigens, and then return the DCs to the subject to *in vivo* stimulate antigen-specific T cells. The T cells should then undergo expansion, migrate to the periphery, and be available for tumor destruction. This has been a clinical paradigm for well over a decade [30], with over 2000 patients treated with such vaccines, and Phase III trials in four different disease sites currently underway [30]. Sipuleucel-T (patient DCs pulsed with prostatic acid phosphatase as an antigen fused to granulocyte-macrophage colony-stimulating factor as an immune stimulant) was the first cancer vaccine approved by the US Food and Drug Administration (FDA) [31]. There are a number of challenges involving DC-based cancer vaccines including production issues necessary for uniformity in phenotype and activity (the realm of good manufacturing practice—GMP) [32]. There are current questions as to what are the optimal means for generating DCs from precursors, as well as how to (or whether to) “mature” the cells, along with their preservation and re-growth after freezing [30]. Additionally, the nature of the loaded antigenic material (source, format, single *vs.* multiple antigens, *etc.*) likely plays a critical role in the final vaccine formulation, but there is no true consensus [33]. As these DCs are usually injected back into a patient, migration of the DCs to lymph nodes is another area of concern [34,35]. Finally, despite the activation of tumor-specific T cells from the DC vaccine, the local tumor microenvironment contributes heavily to immune suppression at multiple levels [36,37,38,39]. This also takes the form of systemic immune suppression [40,41,42,43], possibly driven by TEX [5,26,44,45].

## 3. Exosomes as Potential Vaccine Candidates (All Hands on DEX)

**Figure 2:**
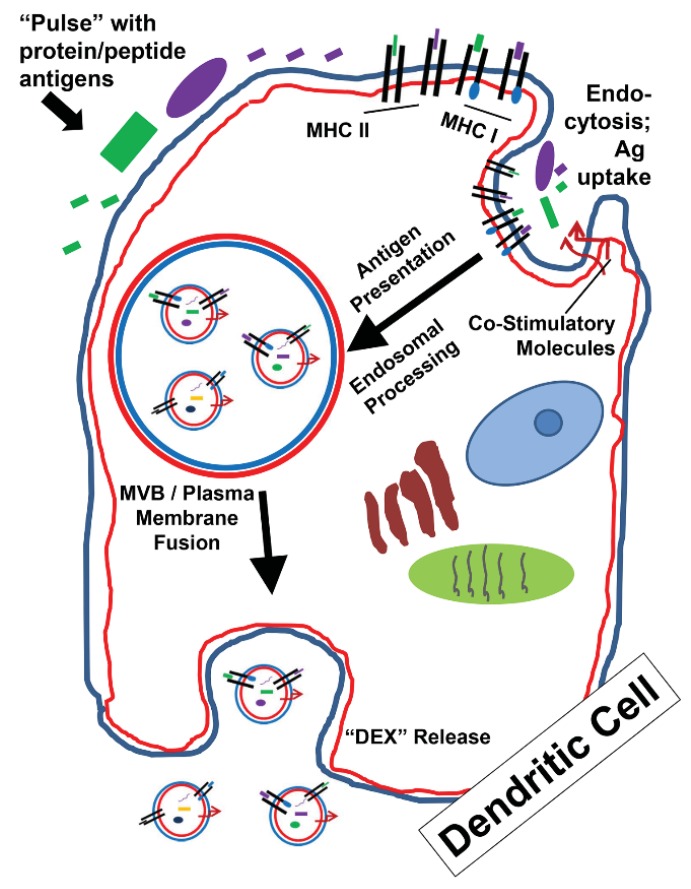
Formation of “DEX” (dendritic cell exosomes, dexosomes). Dendritic cells (DCs) are professional antigen-presenting cells capable of antigen uptake/internalization which leads to presentation of antigens (peptides) on MHC Class I and Class II molecules. This contextual presentation of putative peptide antigens to (respectively) CD8+ and CD4+ T cells requires the addition of co-stimulatory molecules (e.g., CD80/86) to fully mature and activate the T cells. As shown, potentially antigenic proteins (green rectangle and purple oval) and peptides (green and purple small box versions) are loaded (“pulsed”) onto the DCs. Some of the peptides may directly bind to MHC molecules. During aforementioned endocytic events, proteins/peptides, MHC molecules, and co-stimulatory molecules (among other things) are internalized and enter antigen presentation and endosomal processing pathways. Following routes described in Figure 1, MVB formation and the release of DEX lead to extracellular vesicles with MHC molecules and co-stimulatory molecules on their surfaces, along with intracellular proteins that may be antigenic as well.

During the late 1990s/early 2000s, the idea of exosomes as putative cancer vaccines was making headway in pre-clinical and, eventually, clinical settings. This is because exosomes are surrogates of the cells that release them, and perhaps could elicit similar immune responses as could the parental cells themselves. The generation of DEX, dendritic cell exosomes, is shown in Figure 2. As cell-free versions of DC vaccines, one could presumably derive DEX vaccines by collecting the media from antigen-pulsed DCs and isolating the exosomes. In theory, the DEX could likely be stored, thawed, and utilized more efficiently than could the DCs themselves. It was known that professional antigen-presenting cells (APCs) such as B cells could release vesicles capable of antigen stimulation of T cells [46]. Following that publication, DEX from antigen-pulsed DCs were used as cancer vaccines in mice bearing relatively immunogenic (P815) and poorly-immunogenic (TS/A) syngeneic tumors. The anti-tumor responses were presumed to be T cell–dependent (there was no effect on tumor growth if the DEX were utilized in athymic/nude mice models), there were tumor-specific CTL responses generated, and the DEX were perhaps even more effective than the DC vaccines themselves [47]. Follow-up studies included a molecular characterization of DEX, identifying such proteins as MHC Class I and II molecules, co-stimulatory molecules such as CD86/B7.2, potential surface-binding proteins MFGE8, MAC1, and CD9, and an enrichment for the heat shock protein HSC73/HSPA8 [48]. The latter might be regarded as a “danger signal” for innate immune stimulation [49,50,51].

**Figure 3:**
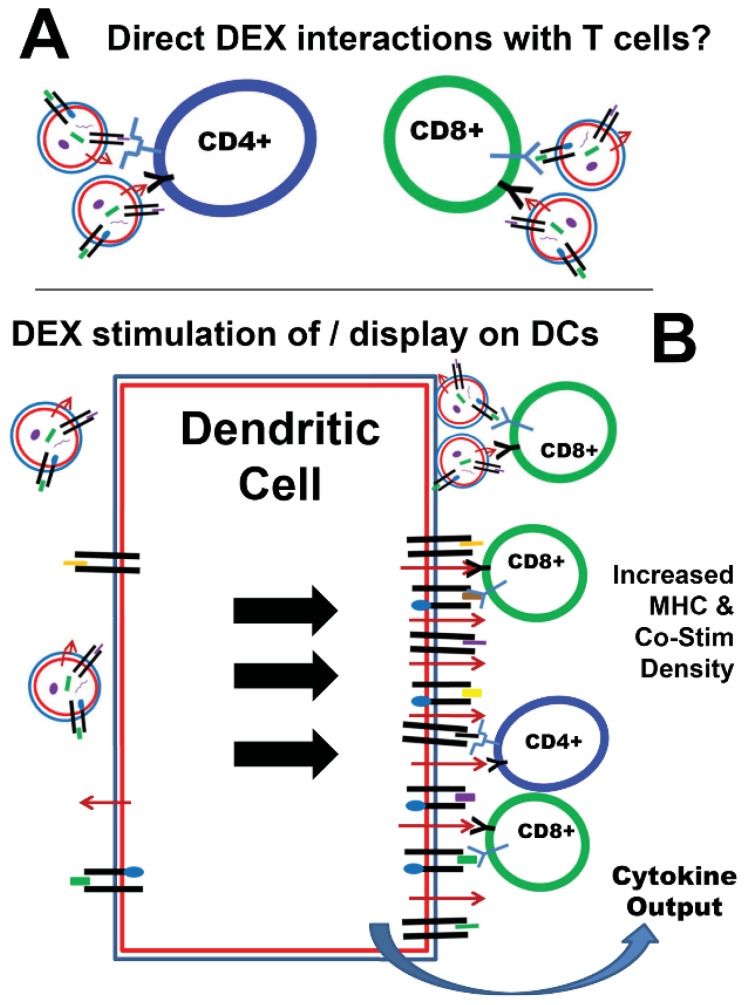
Interactions of DEX with T cells and dendritic cells. (**A**) While there is speculation and some evidence (in an artificial system *in vitro*) that DEX may directly interact with and present antigen to T cells, it is not clear that this happens *in vivo*. Instead, DEX are believed to stimulate DCs (**B**) to cause them to be better antigen-presenting cells (with increased density of surface MHC and co-stimulatory molecules, as well as cytokine output). It is also possible that DEX may adhere to DC surfaces and directly present antigenic peptides in the context of MHC molecules.

As to the mechanism of the DEX-generated immunity, the concept of exosomes as cellular surrogates suggested that DEX would have properties of DCs, and would be able to drive T cell responses in a similar fashion (e.g., antigen presentation and co-stimulation) [47]. Other studies suggested that this was not true, in that cells expressing a specific MHC II with display of an antigenic peptide released exosomes that were poor direct stimulators of T cells, but were effective stimulators if applied to DCs that were then exposed to the T cells [52]. The transfer of the MHC II/peptide complex occurred from the exosomes to the DCs, and it is speculated that there may be a similar transfer of MHC II to follicular DCs by B cells (although these vesicles appear to reside on the follicular DC surfaces). It was speculated that such vesicles may be important in guiding antigen-specific T cells into germinal centers for further B cell development [53]. This was further shown in a system with MHC II–displayed antigen using DCs that lacked MHC II expression; MHC II/antigen complexes were transferred from “wild-type” DCs to the MHC II–deficient DCs via exosomes, and those latter DCs could stimulate T cell-specific responses [54]. Additionally, this was shown to be the mechanism for MHC Class I––displayed peptides via transfer from DEX to endogenous DCs [55]. However, there remains the possibility that DEX can directly stimulate T cells, but this was typically considered a weak response [56]. Similarly, T cell stimulation required specific parameters on the vesicles derived from *Drosophila* cells used as model APCs (high-density display of MHC, presence of co-stimulator B7, and adhesion protein ICAM1), and the *in vivo* results did not rule out interaction with endogenous DCs [57]. Further work demonstrated that membrane vesicles derived from sonicated DCs could directly stimulate T cells, bypassing DC intervention, at least *in vitro*, but again, vesicle-DC interactions could not be ruled out *in vivo* [58]. The concepts of DEX/T cell and DEX/DC interactions are diagrammed in Figure 3.

The use of DEX in tumor vaccine scenarios had been proposed multiple times (there are far more review articles than trial reports) [59,60,61,62,63], and clinical-grade DEX preparations had been described [64], thus leading the way for clinical trials. Results from one such trial [65] for patients with Stage IIIB/IV non-small cell lung carcinoma (NSCLC) treated with DEX loaded with MAGE3/4/10 peptides showed the DEX vaccines were well tolerated, and some patients demonstrated minor immune responses via delayed-type hypersensitivity (DTH) analysis and *in vitro* assays, with more significant responses seen in natural killer (NK) cell activity. While three of the patients had progressive disease prior to DEX treatment, two patients with stable disease incoming were progression-free 12 months after treatment. Another DEX cancer vaccine trial [66] for patients with Stage IIIB/IV metastatic melanoma reported excellent safety profiles, with one objective response, one minor response, and two disease stabilizations noted. The DEX in this case were derived from DCs pulsed with MAGE3 peptides for both MHC I and II, as well as the DEX themselves receiving direct MAGE3 peptide loading. In both of these trials, the lack of potent T cell responses, but the promotion of NK cell numbers and activities [67], suggested that these could be areas for future engagement for DEX vaccines, along with attempts to repress the tumor-induced immune suppression [68]. However, a recent DEX vaccine trial for patients with advanced squamous cell carcinoma of the esophagus, where DEX were obtained from SART1 peptide-pulsed monocyte-derived DCs, did show enhanced T cell responses via ELISPOT assays, although the clinical responses were not spectacular [69]. The modulation of DEX by the ligand expression and maturation state of DCs, by the antigen-loading of DCs, and by the use of chemotherapies to suppress regulatory T cells (Tregs) are strategies employed in an ongoing DEX vaccine trial for patients with NSCLC [70]. These clinical trial results suggest that DEX may be a viable cancer vaccine strategy in terms of feasibility of preparation, ability to deliver multiple doses, and satisfactory safety profiles. As was true of most cancer vaccination scenarios of the times, no outright attempts were made to control or mitigate tumor-induced immune suppression, which will likely play important roles in future cancer vaccine trials [71].

One point of interest that will recur in our analyses of tumor exosome (TEX) vaccines is that B cell involvement may play important roles in anti-tumor efficacy. This was also demonstrated in animal models of DEX vaccines, where DEX from DCs were pulsed with whole ovalbumin (OVA) protein as a model antigen, or with OVA SIINFEKL peptide, which binds to the murine H-2K^b^ MHC I haplotype. Whole OVA protein generated better *in vivo* CD8+ T cell responses, as opposed to the SIINFEKL peptide; those CD8+ T cell responses were both CD4+ T cell- and B cell-dependent [72]. This led to better tumor growth control and increased overall survival of mice challenged with OVA-expressing tumors. In that context, the use of B cell-deficient mice reduced CD8+ anti-SIINFEKL responses (and it was known that CD4+ T cells were essential for the CD8+ activity). The suggestion was that B cell regulation of CD4+ T cells was critically important in optimal CD8+ T cell effector function.

While most research in exosome-based immune effectors centers on T cells and NK cells (and to some extent, B cells), Gehrmann *et al.* [73] looked at the impact of the CD1d display of the ligand α-galactosylceramide (α-GC) on DEX. CD1d is a non-classical MHC-type molecule that presents lipid antigens to effector cells, in this case invariant natural killer T (iNKT) cells (also called Type 1 or classical NKT cells). These cells express an invariant T cell receptor α chain as well as natural killer cell markers, and they respond to glycolipid antigens presented by the CD1 family of molecules. In this study, murine DCs were pulsed with OVA, or with SIINFEKL peptide, and/or α-GC. DEX from those DCs could activate iNKT cells as well as gamma-delta (γδ) T cells (another unconventional T cell family with a restricted T cell receptor repertoire). The combination of these enhanced innate immune effector activities led to increased CD8+ T cell responses and also boosted CD4+ T cell and B cell responses with notable overall anti-tumor activity.

An obvious drawback to DEX vaccine scenarios is the need for autologous DC cultures, which, as mentioned above, have inherent limitations and cannot be truly “off-the-shelf” products. Non-autologous or allogeneic cell-based vaccines have been clinically utilized for some time [74], and one could imagine a similar approach for DEX. Umbilical cord blood DCs loaded with tumor antigens, and DEX from those cells, have been proposed as potentially useful sources for such cell- or DEX-based vaccines [75]. Another extension of this is a system called CELLine 1000 to generate DEX; in one publication [76], a cancer vaccine formulation called chaperone rich cell lysate (CRCL) [77,78,79,80] was used as the antigen source in a murine brain tumor model. Curiously, CRCL has strong proteomic overlaps with exosomes, and has a novel structure that may lead to similar DC responses as TEX [81]. In a similar set of experiments, but using syngeneic DEX from CRCL-pulsed DCs in the same murine brain tumor model, those DEX drove CBL (Cbl proto-oncogene, E3 ubiquitin protein ligase) and CBLB (Cbl proto-oncogene B, E3 ubiquitin protein ligase) signaling with AKT (protein kinase B) and ERK (extracellular regulated kinase) signaling in stimulated T cells [82].

One interesting point behind the putative value of DEX as vaccines may actually involve impacts of DEX directly on tumor cells. The transfer of antigen-based stimulatory and co-stimulatory molecules from DEX to tumor cells would lead to better T cell reactivity against the tumor in ways that are alternative to DC-based stimulation of T or NK cells [83]. This is a novel application of DEX in terms of directly modifying tumor cells to make the tumor more amenable to immune attack.

## 4. TEX as Anti-Cancer Vaccines

### 4.1. Stand-Alone TEX

**Figure 4:**
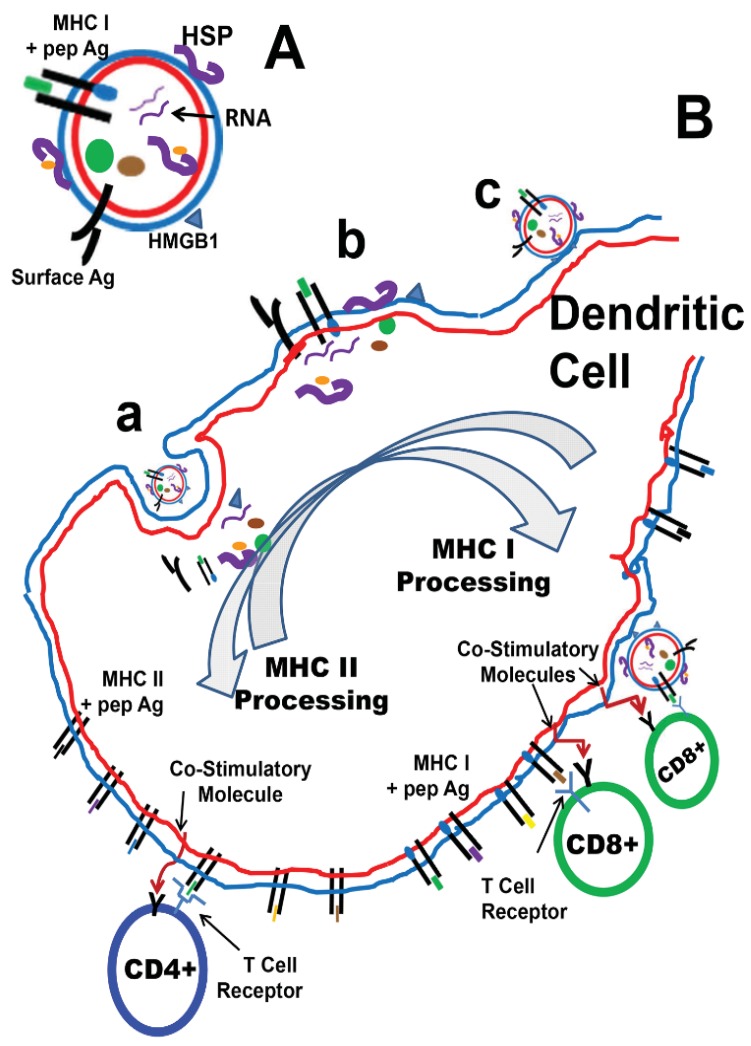
Tumor exosomes (TEX, texosomes) and their interactions with dendritic cells. (**A**) Shows a hypothetical texosome with a potential surface antigen, innate stimulatory molecules such as high mobility group B1 (HMGB1), and heat shock proteins (HSPs, both surface and interior, including those with bound peptides (yellow dot)). Also possibly present might be MHC molecules (generally Class I) with peptide antigens displayed, and other proteins (green and brown ovals) as well as nucleic acids such as RNAs. Potential routes of interactions and transfer or internalization of vesicle contents are shown in (**B**). Part **a** depicts endocytic means of internalization (as mentioned in Figure 1) by numerous mechanisms. Part **b** is the result of direct membrane fusion between TEX and the DC, transferring intravesicle components into the cell interior while depositing vesicle membrane proteins and lipids on/into the DC plasma membrane. Part **c** shows “docking” of the TEX on the surface of the DC without apparent internalization. This would allow stimulus of the DC with presentation of TEX antigens in the context of TEX MHC molecules, as shown on the other side of the cell. Exosome/extracellular vesicle uptake by recipient cells is reviewed here [91]. Processing of the TEX-derived antigens yields TEX peptides on MHC I and MHC II molecules (presenting to CD8+ and CD4+ T cells, respectively), along with enhanced output of co-stimulatory molecules for more effective T cell priming. It is also conceivable that mRNA from TEX could be translated in the DC to generate tumor antigens for presentation as well.

Not long after the pre-clinical DEX cancer vaccine reports were published, the use of TEX was reported as a potent source of immunogenic targets in murine tumor models TS/A, P815, and MC38 [84]—including the ability of TEX from one tumor to cross-prime antigens that led to rejection of the other tumor type (with a different MHC background). This strongly suggested that tumor-derived exosomes contained antigenic material, and could passage common or shared antigens into appropriate antigen presentation pathways of recipient APCs. In the case of the allogeneic cross-protection model, the transfer of presumably membrane-bound MHC/peptide complexes from exosomes to APCs was not considered to be a mechanism, in contrast with what had been shown for some of the DEX studies [52,54,55]. Further, using human melanoma cells as exosome sources, the authors showed the presence of full-length tumor antigen MART1/MLANA, and DCs pulsed with those TEX could present antigens to stimulate MART1-specific T cell clones. TEX-derived antigen delivery and presentation by recipient DCs is shown in Figure 4. A summary of TEX vaccine publications is in Table 1.

After that initial TEX-based vaccination publication, others soon followed, including one utilizing murine plasmacytoma TEX in a vaccination setting that generated protective T cell-based immunity, and demonstrated the presence of known tumor-specific antigens (IAP (intracisternal A particle) *gag*-related structural protein p73, and tumor rejection antigen P1A (Trap1a)) [85]. TEX were clearly important sources of tumor antigens and immune stimulus in several models, including *in vitro* work with human immune cells and human tumor-derived TEX [86]. This article also made a salient point about the source of TEX—for most murine models, one acquired TEX from tumor cell culture-spent medium, which would seem an unlikely source for personalized treatment of human tumors. The authors proposed that ascites fluid (an unfortunate outcome from several cancer types) could serve as sources of TEX for patients afflicted with ascites. However, for tumors that did not have associated ascites accumulation, there are not many obvious biofluid sources of TEX. One potential means around this issue could be to prepare exosome-like nanovesicles from solid tumor sources by sonication of single cell suspensions followed by density-gradient centrifugations [87].

Despite the conflicting evidence as to their *in vivo* immunomodulatory role that began to appear about the same time (see below), TEX have been shown to be potent anticancer vaccines in animal models, driving antigen-specific T cell and B cell responses. Below we highlight some of the works involving unmodified TEX in cancer immunotherapy.

Various publications highlighted the use of TEX as cancer vaccines in animal models, simply using TEX harvested from cell culture–spent supernatants without any modifications or manipulations. This was shown for the leukemia model [88] in a prophylactic setting where increased CTL activity was noted. In an overlap with the heat-stress modalities (see below), it was determined that heat-shocked murine brain tumor cells release heat shock proteins and exosomes that were likely responsible for tumor eradication if those stressed cells were implanted as tumors [89]. Following that, TEX from (unstressed) cells were shown to have HSPs on their surfaces, along with known tumor antigens (EGFRvIII, GPNMB), as well as transforming growth factor beta (TGFB). These TEX were potent vaccines driving T and B cell responses in a prophylactic setting, but had no impact in a stringent (intracranial), pre-established tumor setting [90].

**Table 1:** Summary of TEX as vaccines.

Reference	Tumor Type	TEX (Mod/Engin)	Vax Route	Adjuvant/StiMulus	Outcome	Notes
Wolfers *et al.*, 2001 [84]	TS/A; MC38 (mouse)	TEX only	SC/ID	None	Autologous and allogeneic cross protection DC presentaion of XO Ags	CD4+ and CD8+ dependent
	L1210; AK7; Ba/F3; (mouse)	or TEX on DCs				
	Fon; Mel-888 (human)					
			
			
Altieri *et al.*, 2004 [85]	J558; MPC11; Colon 26 (mouse)	TEX only	SC	None	Prophylactic and rechallenge protection	Tumor-specific
						CTLs active
Bu *et al.*, 2006 [88]	L1210 (mouse)	TEX only	SC	None	Prophylactic protection	CTLs active
Graner *et al.*, 2009 [90]	SMA-560vIII (mouse)				Prophylactic and rechallenge protection	B, T cells active
		
Hao *et al.*, 2006 [92]	EG7/OVA (mouse for Exos)	EG7 TEX; DEX fromOVA-pulsed DCs	IV	None	DEX > TEX *vs.* Metastatic disease (prophylaxis)	DEX > TEX CTL activity
	B16-OVA (mouse tumor target)					
Gu *et al.*, 2015 [93]	WEHI3B; RENCA (mouse)	DEX pulsed with TEX as vax	SC	None	DEX/TEX > DEX/lys for preventing tumor growth/overall survival	Increase CTL and NK activity
Dai *et al.*, 2005 [94]	LS-174T (CEA+, human)	TEX from cells +/− heat shock	SC		HS TEX from CEA+ cells > anti-tumor responses	Increase CD4+/CD8+ activity
	SW480 (CEA+, human)					
	LoVo (CEA+, human)					
	A549 (CEA−, human)					
	(mice txg for HLA-A2.1)					
Cho *et al.*, 2009 [95]	CT26-MUC1; B16-MUC1 (mouse)	TEX from cells +/− heat shock	ID	CPG (anti-tumor) IFA (for Abs)	HS TEX > auto/allogeneic anti-tumor responses	HS TEX > B and T cell responses
Xie *et al.*, 2010 [96]	J558 (mouse)	TEX from cells expressing HSP70 on surfaces	SC	None	TEX70 > TEXhs > TEX in anti-tumor assays	IncreaseCD4+, CD8+, NK responses
Chen, *et al.*, 2011 [97]	Lewis lung carcinoma (3LL, mouse)	TEX from cells +/− heat shock	IT, SC	none	HS-TEX > TEX in anti-tumor assays	HS-TEX contain chemokines, attract DCs and T cells
Yang, *et al.*, 2007 [98]	EG7/OVA [IL2 tfxt for TEX] (mouse)	TEX w/IL2	SC	none	TEX-IL2 > TEX + IL2, TEX in anti-tumor assays	CD8+ > CD4+ > NK effectors
Xie *et al.*, 2010 [99]	J558 (mouse, P1A Ag) tfxt to express TNFA, IL2, IFNG	TEX from each transfectant	IV	none	TEX/TNFA > TEX.IL2 > TEX/INFG in anti-tumor assays	Same order for P1A-specific CTL
Lee *et al.*, 2011 [100]	B16F1 (mouse)[CIITA tfxt for TEX]	TEX w/CIITA	ID	none	TEX/CITTA > TEX in in anti-tumor assays	TEX/CITTA > TEX for DC, T cells, B cells
Rountree *et al.*, 2011 [101]	CT26-PAP; E6-PSA (mouse, expressing human Ags)	Immunize w/virus to drive PAP or PSA exo expression linked to C1C2 lactadherin domain	SC (virus)	none	Ag/C1C2 > untargeted Ag in anti-tumor assays	Similar responses in B cell and T cell assays; B cells were strain- dependent
Zeelenberg *et al.*, 2011 [102]	MCA101-OVA (mouse)	cells tfxt for soluble OVA, membrane OVA, or TEX-OVA (via C1C2)	cyroablation	none	TEX-OVA > sOVA ≥ fcOVA tumors for immune activation and anti-tumor response	
Sedlik *et al.*, 2014 [103]	MCA101-OVA, EL4-OVA, B16F-OVA (all mouse, expressing OVA)	cells tfxt for gag-OVA or C1C2-OVA (DNA vaccine)	ID, IM electroporation	none	both vax had ~ equal benefit in anti-tumor and cellular responses	
Hartman *et al.*, 2011 [104]	4T1--HER2 (mouse, human HER2)	C1C2-CEA, C1C2-HER2 (ECDs fused to C1C2) AdVir vaccine	ID	none	C1C2-HER2 > ECD-HER2 in anti-tumor responses	C1C2-Ag > ECD-Ag for B and T cell responses
Zeelenberg *et al.* 2008 [105]	MCA101-OVA (mouse)	C1C2-OVA, solb OVA (DNA vaccine)	IM	none	C1C2-OVA > sOVA in anti-tumor response	C1C2-OVA > sOVA in T cell responses
Xiu *et al.*, 2007 [106]	EG7 (OVA) (mouse)	TEX with SEA or TM-SEA “transfer”	SC	none	TEX/TM-SEA > TEX/SEA > TEX > SEA in anti-tumor responses	TEX/TM-SEA > TEX/SEA > TEX > SEA in T cell assays
Dai *et al.*, 2008 [107]	CRC with ascites Stage III−IV (human) CEA+ in sera	AEX (TEX from ascites)	SC	some GM-CSF; various chemos	1 pt w/stable disease 1 pt w/minor response	AEX+GM-CSF > AEX for DTH and anti-CEA T cells

Table 1. Summary of TEX as cancer vaccines. Abbreviations: OVA = ovalbumin; TEX = tumor-derived exosomes; SC = subcutaneous; IM = intramuscular; ID = intradermal; IV = intravenous; IT = intratumoral; DC = dendritic cells; XO = exosomes; IFA = incomplete Freund’s adjuvant; IL2 = interleukin 2; INFG = interferon gamma; TNFA; tumor necrosis factor alpha; CIITA = class II major histocompatibility complex, transactivator; ECD = extracellular domain; SEA = staphylococcal enterotoxin A; TM = transmembrane; GM-CSF = granulocyte-macrophage colony-stimulating factor; PAP = prostatic acid phosphatase; PSA = prostate-specific antigen; CRC = colorectal cancer.

In a comparison between DEX and TEX as forms of immunotherapy, Hao *et al.* [92] used EG.7 cells (OVA-expressing tumor as a model antigen) as sources of TEX vaccines, and compared immune parameters to mice immunized with DEX from OVA-pulsed DCs. DEX drove greater *in vitro* expansion and cytotoxicity of T cells from treated mice, as well as better overall anti-tumor immunity, but that effect was seemingly reliant on OVA as the antigen (the *in vivo* tumor model was a different OVA-expressing tumor). Other tumor-specific antigens from EG.7 would likely not be relevant in the B16-OVA model used. B cell/antibody induction did not appear to be measured. Curiously, the exosomes were delivered intravenously (IV), which likely impacts how and which immune cells encounter the exosomes (see below).

Rather than injecting TEX directly for presumed APC interaction, TEX have been used as immunogens for DC loading. Using murine leukemia and renal cell cancer models, TEX as immunogen sources proved superior to tumor lysate for promoting DC-driven anti-tumor immunity. TEX-loaded DCs showed persistence of TEX (and presumably TEX products) in DC MHC II-containing compartments. Those DCs provoked more trogocytosis (exchange of DC surface materials to T cells). The persistence and localization of TEX/TEX material within DCs were suggested as mechanisms for the improved anti-tumor efficacy [93].

### 4.2. TEXing while Driving Immune Responses: Manipulations of Cells to Produce TEX with Enhanced Immune Properties

Manipulation of the cells producing TEX could potentially lead to TEX that are more efficient at driving immune responses. This has been configured in multiple ways and several examples are illustrated below.

#### 4.2.1. Heat Shock, Cell Stress, HSPs, and TEX Effects

One means of potentially generating enhanced immune responses is to increase “danger signal” expression via increased heat shock protein expression. As noted above and elsewhere [108,109], HSPs, particularly those exposed on cell surfaces or released extracellularly, can ameliorate innate immune responses, leading to heightened adaptive responses. Dai *et al.* [94] heat stressed murine tumor cells expressing human carcinoembryonic antigen (CEA) in a transgenic (human MHC) model, demonstrating that TEX from the heat-shocked cells drove better anti-tumor immunity compared to TEX from untreated cells. In another setting [95], TEX from heat-shocked, MUC1-expressing tumors of different MHC haplotypes (CT26 and B16) were better than control TEX at inducing anti-tumor immune responses in both syngeneic and allogeneic settings (*i.e.*, treating mice with TEX from heat-shocked CT26 cells to reject B16 tumors). HSPs, especially HSP70, were considered to be important players in the Th1 polarization of lymphocytes. This paper also demonstrated increased antibody output against the injected exosomes, along with [90], among the first to point this out. In another iteration, the group utilized an engineered membrane HSP70 that localized to TEX surfaces (along with the P1A tumor antigen in a myeloma model). These TEX were taken up by DCs, which in turn matured and were effective at inducing CTL responses against P1A-expressing tumor cells (TEX and DC + TEX were also used as prophylactic vaccines). It was found that NK cells played prominent roles in the anti-tumor response [96]. Furthermore, TEX from heat-stressed Lewis lung carcinoma cells proved effective at attracting and activating DCs, along with T cells, and drove profound tumor regression upon intratumoral injections. The HS TEX contained the chemokines CCL2, 3, 4, 5, and 20, which associated with lipid raft components following hyperthermia treatments [97]. Further speculation suggests that either whole-body hyperthermia or localized treatments may lead to TEX release as another form of “self-vaccination” [110].

Chemotherapy agents also induce stress programs, such as the heat shock response [111], which ordinarily favor tumor survival. However, TEX displaying surface HSPs following drug treatment of hepatocellular carcinoma cells were potent stimulators of NK cell activation and cytotoxicity *in vitro* [112]. This suggests that chemotherapy may be able to induce a “self-vaccination” phenomenon with TEX HSPs as NK cell triggers. Similar results were obtained treating hepatoma cells with a histone deacetylase inhibitor [113]. On the other hand, some speculate that such TEX could potentially serve as decoys, leading to ineffective NK cell responses (including downregulation of activation receptors and upregulation of inhibitory receptors) [114].

#### 4.2.2. Engineering the Parent Cells—The TEX of New Immune Ideas

Modification of tumor cells to make them more immunogenic and therefore potentially useful vaccine material has long been a goal in cancer immunotherapy [115]. The concept of using TEX from modified tumor cells as surrogate vaccines was a logical next step. Genetic introduction of interleukin 2 (IL2) into EG.7 (ovalbumin-expressing) tumor cells resulted in the presence of IL2 in (or on) TEX from those cells. When used as vaccines in both prophylactic and pre-established tumor settings, the IL2-containing TEX were more effective than unmodified TEX or TEX mixed with IL2; CD4+, CD8+, and NK cells were all involved in the anti-tumor immunity [98]. To perhaps better ensure membrane expression of IL2, T24 human bladder cancer cells (expressing the tumor antigen MAGE1) were transfected with a construct that linked IL2 to a glycosyl-phosphatidylinositol (GPI) anchor resulting in membrane display on the cells and apparently on the TEX as well. These GPI-IL2 TEX stimulated DCs more efficiently to generate specific CTL responses against T24 cells *in vivo* [116].

In a more extended version of immune gene manipulation of tumor cells, the aforementioned P1A-expressing myeloma cells were transduced to express TNFA, IL2, or IFNG, and TEX from those cells (and unmodified controls) were used in an IV vaccination scenario. TEX from TNFA-transduced cells were superior in both tumor protection and increasing P1A-specific CTLs [99].

To induce increased CD4+ T cell responses (hopefully leading to better CD8+ T cell responses), one group transduced murine B16 cells with the Class II transactivator (CIITA) gene, leading to higher MHCII display on TEX. Pulsing DCs with CIITA TEX strongly activated DCs, resulting in better T cell and B cell activation *in vitro* and *in vivo*, along with improved anti-tumor activity (intradermal vaccination) [100].

Further use of engineered cells producing TEX as a means of antigen loading of DCs involved the use of a lymphoblastoid “cell factory” transfected with HER2 (ERB2/EGRF2). TEX from those cells efficiently transferred ERB2 peptides to MHC I and MHC II displayed on DCs that could stimulate CD8+ responses from cells in the blood of ERB2+ cancer patients [117].

#### 4.2.3. Exosome Display—Filling the TEX Box

Genetic manipulation of parental cells to enhance TEX immunogenicity relies on the possibility that such cellular alterations lead to changes in the TEX. The concept of “exosome display technology” [118] employs directed targeting of potential tumor antigens for incorporation into exosomes as cargo. In one example, a vaccinia virus construct was used to fuse prostate-specific antigen (PSA) or prostatic acid phosphatase (PAP) to the C1C2 (exosome-targeting) domain of lactadherin. Mice prophylactically immunized with the exosome-targeted versions of the prostate cancer antigens had better anti-tumor responses (including activated T cells and serum antibody responses) than did mice immunized with the same vaccinia platform where the antigens were not specifically exosome-targeted [101]. Exactly what cell types, and which exosomes from the presumably immunized cells, provoked the immune responses were not determined. These results were recapitulated to some effect in a setting where OVA tumor antigen localization was controlled to secreted, membrane (cell)-bound, or TEX-targeted. Vesicle-targeting of OVA again resulted in prominent antibody and T cell responses leading to greatly reduced tumor growth and activated CD8+ T cells (following tumor cryoablation) [102]. Other versions of “exosome display” targeted OVA via a DNA plasmid vector vaccine to either exosome external surfaces (via lactadherin C1C2 domain) or to the interior surfaces (via a viral Gag construct) [103]. From an overall tumor rejection perspective (vaccinations done by intramuscular and/or intradermal injection and electroporation), there were essentially no differences between the two constructs, but the C1C2 linkage generated more active CD4+ T cells, and an IgG1 *vs.* IgG2b shift in antibody output, at least at lower vaccine dosages.

Using a different vector, adenovirus constructs with extracellular domains of CEA or HER2 targeted to exosomes (via lactadherin C1C2 domain) again produced specific T cell and antibody responses, to some extent even in antigen-tolerant animal models, with subsequent improved tumor rejection in the HER2 model [104].

Another report pursued engineered membrane expression of OVA (in a murine MCA (methylcholanthrene-induced) sarcoma model) that led to its localization in exosomes as cargo [105]. The presence of the antigen in vesicles in the membrane-bound form resulted in a lymphocyte-dependent reduction in tumor growth kinetics, as if the tumor could “self-vaccinate”.

#### 4.2.4. Altering TEX Directly—New Immune Fonts

Another means of modification would be to directly alter the TEX. In one instance [106], superantigen staphylococcal enterotoxin A (SEA) was physically tailed with a hydrophobic segment that allowed its incorporation into exosomes from EG.7 tumor cells, presumably as a transmembrane protein (SEA-TM). As prophylactic vaccines, these SEA-TM TEX proved to be more effective CD8+ inducers and were overall better in reducing tumor burden than SEA-modified TEX or parental EG.7 TEX.

#### 4.2.5. Clinical TEX: Use in a Clinical Cancer Vaccine Trial

As DEX had been used in clinical trials (see above), TEX cancer vaccines were expected to follow suit, including a position paper essentially describing the putative combination of chemotherapies and immune agonists for use in treating patients with ovarian cancer [119]. Following the aforementioned use of ascites fluid–derived TEX as sources of autologous antigens, early works demonstrated the presence of known tumor antigens from TEX collected from various malignant effusions, ascites in particular, that could be presented by DCs to specific T cells [120]. A guide for clinical-grade TEX preparations was developed, along with suggestions for adjuvants [121], but nothing seemingly matriculated for the next couple of years. Eventually there was a clinical trial performed in China [107] where TEX were harvested from ascites fluid of 40 patients with colorectal cancer (the TEX were termed AEX) and were used for autologous vaccinations with or without GM-CSF. Patients were of HLA-A*0201 haplotype, and had CEA detectable in sera. Patients were subcutaneously injected with 100, 200, 300, or 500 μg of AEX for a total of four vaccinations, and half of the patients received 50 μg of GM-CSF per vaccination. Patients all had DTH responses at doses of 300 μg AEX or greater, but required GM-CSF for significant differences at 200 μg AEX. Using CD8+ T cells isolated from the DTH sites, tetramer responses (*vs.* CEA), along with IFNG release and cytotoxicity driven by CEA+ cells, were significantly increased in patients receiving AEX + GM-CSF. Safety and tolerability were very good, although no outstanding clinical responses were noted. To our knowledge, these are the only published clinical results from a TEX-based cancer vaccine trial. The trends of TEX in immune responses had shifted from TEX as immune stimulators to TEX as immune suppressors, and we will examine some of those studies in the next section.

## 5. TEX in Immune Suppression

TEX certainly have immune-suppressive attributes, and these have been reviewed extensively [4,44,114,122,123,124,125]. Here, we will briefly discuss some aspects of TEX immunosuppression as it relates to T, B, and NK lymphocytes as well as monocytic cells.

### 5.1. “Bad” TEX: Vessels of Tumor-Induced Immune Suppression of Lymphocytes and Monocytes

#### 5.1.1. T Lymphocytes

Around the same time that TEX were considered to be cancer vaccine candidates, further examination was finding that TEX had immune suppressive qualities as well, including the ability to promote induction of apoptosis and signaling defects in lymphocytes [8,126,127]. The apoptotic effects were likely FAS ligand-driven [128], and possibly PD1-L driven [129], but this is not seen in all cases [26]. The effects of TEX surface CD39 and CD73 lead to an ectonucleotidase cascade that generates extracellular adenosine, which has suppressive T cell effects [130]. TEX have also been shown to impair lymphocyte responses to IL2 [131], and to promote activation and expansion of regulatory T cells (Tregs) that are themselves immune suppressive [132]. Again, this is not universally seen [26].

#### 5.1.2. B Lymphocytes

While there is not an extensive literature on TEX effects on B cells, circulating TEX can conceivably interact with therapeutic antibodies injected into patients based on surface-displayed antigens, which can reduce antibody-dependent cellular cytotoxicity (ADCC) [133]. TEX from B cell lymphomas display CD20, and thus shield target cells from the therapeutic antibody rituximab; these TEX can fix and consume complement to prevent complement-dependent cytotoxicity (CDC) as well [134]. Given that TEX are potent generators of serum antibodies, and are known to display numerous tumor-related antigens [90,135], it is possible that TEX could be substantial decoys for exogenous or endogenous anti-tumor antibodies that could essentially titrate out such immune effector molecules.

#### 5.1.3. NK Cells

The presence of NK cell (NKG2D) ligands such as MICA, MICB, and ULBP1/2 on TEX has been known for some time, and these ligands appear to be involved in TEX-dependent suppression of NK cell activity [136]. TGFB, known to be present on TEX [90], seems to be a major culprit in that suppression [137,138]. One line of thinking is that the NKG2D ligands may downregulate the receptor on NK cells, thus reducing NK activity, or with such TEX acting as circulating decoys [139]. On the other hand, the presence of the inducible HSP70 on TEX surfaces is thought to be stimulatory to NK cells, resulting in anti-tumor activity [112,140,141]. It begs the question whether HSP70 as a surface ligand for NK cells could also make such TEX decoys against NK activity.

#### 5.1.4. Monocytes, Macrophage, Dendritic Cells

**Figure 5:**
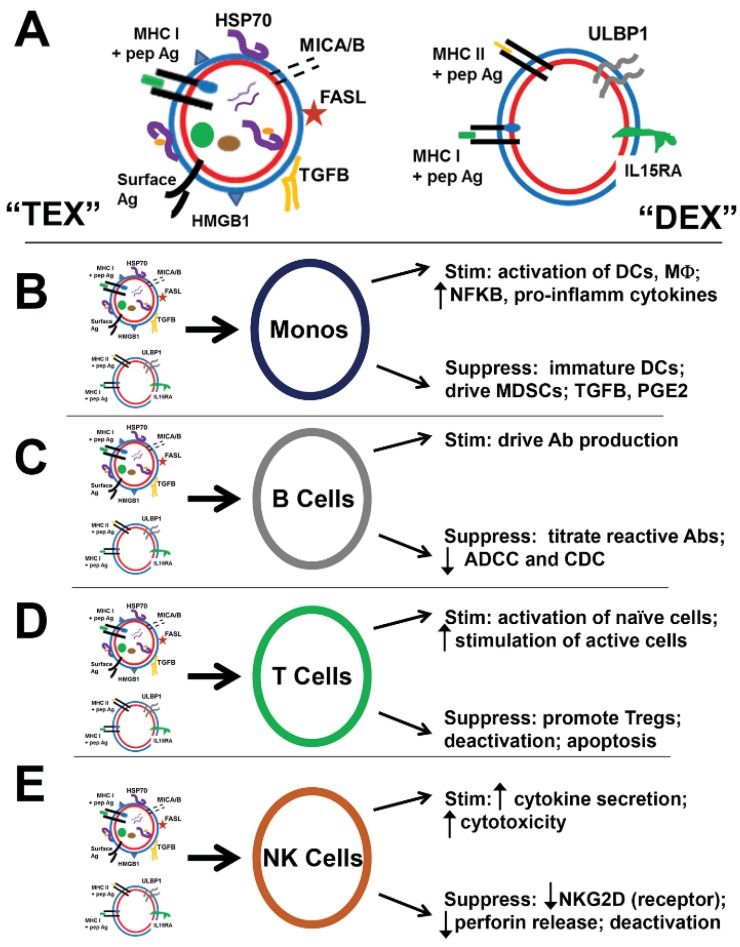
Summary of TEX and DEX interactions with immune cells. (**A**) depicts more extensive versions of TEX and DEX than previously shown (e.g., Figure 2, Figure 3 and Figure 4), emphasizing NKG2D ligands MICA/B and ULBP1, and suppressive entities FASL and TGFB (on TEX), and stimulatory molecule IL15RA (IL-15 receptor alpha, on DEX). The rest of the figure shows possible (and perhaps conflicting) outcomes of TEX and DEX interactions with various immune cells. TEX and DEX interactions with monocytes (“Monos”), shown in (**B**), lead to a variety of outcomes depending on the recipient cells. Immature dendritic cells (DCs) may be activated to further maturation, resulting in immune (T cell) stimulation, or may be held in an immature state, which is largely considered suppressive. Macrophages (MФ) are often stimulated, producing pro-inflammatory cytokines via activation of NFKB. However, TEX in particular may interact with undifferentiated monocytes to push them (often via TGFB) into a monocyte-derived suppressor cell (MDSC) phenotype, producing immune suppressive factors TGFB and PGE2. B cells (**C**) may interact with TEX and DEX in ways that stimulate antibody (Ab) production; however, TEX are able to bind serum Abs via surface antigens and can titrate potentially tumor-reactive Abs out of the system. This results in reduced antibody-dependent cellular cytotoxicity (ADCC). Further, TEX can bind complement components and prevent complement-dependent cytotoxicity (CDC). As mentioned throughout the text, TEX and DEX interactions with T cells (**D**) may be stimulatory, activating naïve T cells (via DEX, although this is controversial), and possibly further stimulating previously activated T cells (via TEX). However, most TEX/T cell interactions are thought to be immune-suppressive, leading to generation of regulatory T cells (Tregs), anergic and deactivated phenotypes, and sometimes apoptosis, likely driven by FAS/FASL interactions. DEX interactions with natural killer (NK) cells (**E**) via the NKG2D ligand ULBP1, promoted by IL15RA, stimulate NK cells for pro-inflammatory cytokine secretion and enhanced perforin- and granzyme-mediated cytotoxicity. Interactions with TEX, however, are less clear; NKG2D ligands such as MICA/B, and TEX-surface HSP70 (interacting with NK cells via CD94) could be stimulatory, but often leading to down-regulation of NKG2D. TGFB also has suppressive influences resulting in decreased perforin release and general NK cell deactivation.

TEX are known drivers of myeloid-derived suppressor cells (MDSC), monocytic cells of myeloid lineage that regulate functions of DCs, macrophages, T and NK cells [125]. TEX can block differentiation of myeloid precursors [142] and drive MDSCs from those cells, again via effects of TGFB, and also prostaglandin E2 (PGE2) [143]. While TEX-surface HSP70 was considered stimulatory to NK cell activity (above), it seems to lead to generation of MDSC by TLR2/MyD88 stimulation and activation of STAT3 [144]. On the other hand, IV-injected TEX targeted to brain/lung macrophages, and upregulated pro-inflammatory cytokines via NFKB activation through TLR2 stimulation. Palmitoylated proteins on TEX surfaces led to NFKB activation [145]. This may or may not conflict with the induction of monocyte/macrophage M2 phenotypes (considered more tumor-promoting) by glioma TEX [146], but B16 TEX were regarded as inducers of DC maturation and CD4+ T cell stimulation, driving NFKB activation in RAW264.7 macrophage cells; however, those cells released a mixed bag of cytokines/chemokines with overall Th2/M2 phenotypes [147]. A summary of TEX (and DEX) interactions with various immune system cells is shown in Figure 5.

In addition to their immunosuppressive roles, TEX have been shown, in several cancer models, to actively promote tumorigenesis and metastases. Mechanisms for these actions include modulation of bone marrow progenitors, modification of sentinel lymph nodes, and transfer of oncogenic material including receptor proteins and RNA [148,149,150]. TEX have been shown to help establish the pre-metastatic niche through the creation of a suitable microenvironment for metastatic growth in distant sites [151]. Thus, while related to (suppressed) immunity, other functions of TEX benefit tumor growth and metastasis as well.

## 6. Immune Stimulation or Immune Suppression; Is It All in the ConTEXt?

From the preceding reports, it is clear that TEX both support and suppress immune responses against tumors. There is the obvious presence of tumor antigens and other immune-stimulatory molecules (e.g., HSPs) in and on TEX, and their virus-like particle character [152,153] suggests that exosomes could be useful vaccines, and this is borne out of the literature in this article. In other circumstances, preparation of nano-vaccines based on exosome characteristics should also be feasible [154,155,156]. On the other hand, the immune-suppressive features are also evident. Many of the challenges in determining which direction the immune response goes lie in *in vivo* systems that are, by definition, complicated. One thing that does not often receive sufficient attention is the route of injection (generally in animal models) leading to exosome trafficking. Most of the reports herein used subcutaneous or intradermal injections; we have noted above instances where other routes (intravenous, intratumoral) were used.

This has been reviewed recently to some extent, but has mostly focused on DEX and B cell-derived exosomes [157], which may influence which recipient cells encounter the exosomes and may also determine the responses to those exosomes. However, some principles likely apply, such as the rapid clearance of exosomes by the hepatic and splenic reticuloendothelial system (RES) following intravenous injection. This would be predicted based on known biodistribution of liposomes and other nanoparticles [158], but one might consider the possibility that appropriately stimulatory vesicles might be able to induce an effective immune response via the phagocytic APCs of the RES. On the other hand, as noted in [157], trafficking studies essentially rely on bulk exosome availability for detection; small exosome quantities would not be noticed in most cases. Thus, interactions with lymphocytes in the blood might escape notice, but would seem very likely in a tumor setting where there would be nearly continual release of exosomes into the blood, and at potentially high levels [26].

Intradermal/subcutaneous delivery seems to lead to uptake by CD11c+ DCs that eventually migrate to the draining lymph node [159]; another model found that CD8+ DCs were exosome recipients via the integrin LFA-1 (CD11a/CD18) [160]. These studies imply that dermal APCs are potentially extremely important in the initiation of TEX-induced immune responses leading to T cell activation.

The status of those APCs prior to vaccination is undoubtedly important, and may reflect a number of parameters such as overall stimulatory capacity of the injected TEX, quantity of TEX, and frequency of administration, *etc.* One critical parameter of TEX vaccination may be the systemic immune condition at that time. As mentioned above, if TEX are already in circulation, much data suggests there could be significant lymphocyte dysfunction [161]; this would likely abrogate effects of local APC stimulation of TEX vaccination (e.g., comparing the prophylactic immune response *vs.* that in the pre-established tumor setting [90]). Thus, one would preferably target skin/dermal APCs, but in the context of low or no tumor-induced immune suppression, locally or systemically. This situation may exist after tumor excision or by bulk reduction with chemo/radiotherapeutics, as that should remove the source of the immune-suppressive moieties. This would also depend on the half-lives of those immune-suppressive moieties. Some models, particularly those using exosomes as almost a form of immune accountancy (stimulus or deficit), may help to predict the status of immune activation *vs.* immune tolerance [162] or to determine states in the framework of a cancer-immunity landscape [163]. These would suggest that removal of tumor exosomes from blood would represent a parameter change that could allow for enhanced immune responses, and such devices are currently in a clinical trial [164] (NCT02215902, ClinicalTrials.gov).

Practical issues such as TEX sources may become important; as mentioned above, many tumors do not produce ascites fluid, which seems to be the current choice of autologous TEX. One may consider cell line sources of TEX; allogeneic cell lines as cancer vaccines exist and are in clinical trials [74,165]. As long as sufficient shared tumor antigens are present, along with relatively low quantities of immunosuppressive agents (e.g., TGFB), the allogenicity may actually prove beneficial. Engineering of cells producing TEX, and of TEX themselves (as mentioned above), could yield GMP-quality, off-the-shelf vaccine candidates relevant for numerous tumor types. With FDA approval of immune checkpoint inhibitors [166], potential combination therapies targeting both activation of immune stimulation and suppression of immune suppression are now viable modalities. The TEXual revolution may just be starting.

## 7. Conclusions

Exosomes are important mediators and regulators of immunity for which there is potential widespread therapeutic application. Although TEX natively immunosuppress and facilitate tumor progression, they are also important sources of tumor antigens, with potential clinical application in immune stimulation. This is the dichotomy of TEXual responses in cancer immunity. Initial studies in murine models show promising clinical responses to dendritic cell vaccines derived from TEX as opposed to other antigen sources. However, while preclinical studies show anti-tumor efficacy, in clinical trials the observed immunogenicity of such vaccines remains underwhelming in several studies. Various co-stimulatory molecules have been discovered, such as heat-shock proteins, which may have a further role to play in developing the optimal anticancer vaccine. In addition, some vaccine structural experiments demonstrate differences in efficacy based on location and presentation of the vaccine antigens. It behooves future research to focus not only on development of tumor-derived exosome antigens for cancer vaccines but also to optimize the context in which they are presented to maximize the clinical response. Inhibition of immune suppression will also play a critical role in the development of TEX-based cancer vaccines, which may include preventing TEX effects on immune responders such as T and NK lymphocytes, but will focus TEX effects on APCs. Defining that conTEXt will be critical.
